# Real-world use of multigene signatures in early breast cancer: differences to clinical trials

**DOI:** 10.1007/s10549-023-07227-0

**Published:** 2024-01-24

**Authors:** Luca Licata, Rita De Sanctis, Andrea Vingiani, Deborah Cosentini, Monica Iorfida, Elena Rota Caremoli, Isabella Sassi, Bethania Fernandes, Andrea Gianatti, Elena Guerini-Rocco, Claudia Zambelli, Elisabetta Munzone, Edda Lucia Simoncini, Carlo Tondini, Oreste Davide Gentilini, Alberto Zambelli, Giancarlo Pruneri, Giampaolo Bianchini

**Affiliations:** 1 Department of Medical Oncology, IRCCS Ospedale San Raffaele, Via Olgettina 60, 20132 Milan, Italy; 2 School of Medicine and Surgery, Vita-Salute San Raffaele University, Milan, Italy; 3 Department of Biomedical Sciences, Humanitas University, Pieve Emanuele, Italy; 4 Medical Oncology and Hematology Unit, IRCCS - Humanitas Research Hospital, Rozzano, Milan, Italy; 5 Deparment of Pathology and Laboratory Medicine, Fondazione IRCCS Istituto Nazionale Dei Tumori, Milan, Italy; 6 School of Medicine, University of Milan, Milan, Italy; 7 Medical Oncology Unit, ASST Spedali Civili of Brescia, Brescia, Italy; 8 Division of Medical Senology, IEO, European Institute of Oncology IRCCS, Milan, Italy; 9 Oncology Unit, ASST Papa Giovanni XXIII, Bergamo, Italy; 10 Pathology Unit, IRCCS Ospedale San Raffaele, Milan, Italy; 11 Department of Pathology, IRCCS - Humanitas Research Hospital, Rozzano - Milan, Italy; 12 Department of Pathology, ASST Papa Giovanni XXIII, Bergamo, Italy; 13 Division of Pathology, IEO, European Institute of Oncology IRCCS, Milan, Italy; 14 Department of Oncology and Hemato-Oncology, University of Milan, Milan, Italy; 15 Pathology Unit, ASST Spedali Civili of Brescia, Brescia, Italy; 16 SSVD Breast Unit, ASST Spedali Civili of Brescia, Brescia, Italy; 17 Breast Surgery Unit, San Raffaele Hospital, Milan, Italy

**Keywords:** ER+/HER2− early breast cancer, Adjuvant therapy, Multigene assays, Oncotype DX

## Abstract

**Purpose:**

In Italy, Lombardy was the first region to reimburse multigene assays (MGAs) for patients otherwise candidates for chemotherapy. This is a real-world experience of MGAs usage in six referral cancer centers in Lombardy.

**Methods:**

Among MGAs, Oncotype DX (RS) was used in 97% of cases. Consecutive patients tested with Oncotype DX from July 2020 to July 2022 were selected. The distribution of clinicopathologic features by RS groups (low RS: 0–25, high RS: 26–100) was assessed using chi-square and compared with those of the TAILORx and RxPONDER trials.

**Results:**

Out of 1,098 patients identified, 73% had low RS. Grade and Ki67 were associated with RS (*p* < 0.001). In patients with both G3 and Ki67 > 30%, 39% had low RS, while in patients with both G1 and Ki67 < 20%, 7% had high RS. The proportion of low RS in node-positive patients was similar to that in RxPONDER (82% vs 83%), while node-negative patients with low RS were significantly less than in TAILORx (66% vs 86%, *p* < 0.001). The distribution of Grade was different from registration trials, with more G3 and fewer G1 (38% and 3%) than in TAILORx (18% and 27%) and RxPONDER (10% and 24%) (*p* < 0.001). Patients ≤ 50 years were overrepresented in this series (41%) than in TAILORx and RxPONDER (31% and 24%, respectively) (*p* < 0.001) and, among them, 42% were node positive.

**Conclusions:**

In this real-world series, Oncotype DX was the test almost exclusively used. Despite reimbursement being linked to pre-test chemotherapy recommendation, almost 3/4 patients resulted in the low-RS group. The significant proportion of node-positive patients ≤ 50 years tested indicates that oncologists considered Oncotype DX informative also in this population.

**Supplementary Information:**

The online version contains supplementary material available at 10.1007/s10549-023-07227-0.

## Introduction

More than 90% of breast cancer patients are diagnosed with early-stage disease, and around 70% of them have tumors that are estrogen receptor positive (ER+) and human epidermal growth factor receptor 2 negative (HER2-) [1, 2]. In these cases, adjuvant endocrine therapy is typically recommended as it reduces the risk of recurrence by almost half and decreases breast cancer mortality by a third, with a generally favorable risk–benefit ratio [3].

Adjuvant chemotherapy further reduces the risk of recurrence and death from breast cancer, and the addition of chemotherapy to adjuvant endocrine therapy is indicated in those patients for whom the estimated residual risk despite endocrine therapy is significant [4, 5]. The classical clinicopathologic variables are essential for clinicians to estimate the risk of disease recurrence, but they are of limited use in predicting chemotherapy benefit for individual patients [6].

Advances in the understanding of the molecular biology of breast cancer in the last two decades have led to the development of multigene assays (MGAs) that provide prognostic information independent of that provided by standard clinicopathologic features and help clinicians to better identify those patients with low-risk disease who can be safely spared chemotherapy [7]. All the commercially available MGAs for ER+/HER2 − early breast cancer have been robustly clinically validated [8] and a plethora of studies consistently showed that their use can decrease chemotherapy recommendation in up to 50% of cases [9–17]. Accordingly, major guidelines recommend the use of MGAs as a tool to tailor adjuvant chemotherapy decision [5, 18].

Based on this striking evidence, in July 2019, Lombardy was the first Region in Italy to reimburse genomic testing for patients with ER+/HER2- breast cancer [19]. Subsequently, on May 2021, the Italian National Health System has approved reimbursement countrywide.

In this study, the Lombardy Genomic Assays for Breast Cancer Working Group reports the pilot experience of six referral cancer centers with the use of MGAs after reimbursement.

We aimed to evaluate the clinicopathologic parameters of the patients who were tested and to compare our real-world data with those from the landmark TAILORx and RxPONDER trials.

## Materials and methods

### Eligibility criteria for genomic testing

Starting from July 2019, patients in Lombardy were considered eligible for reimbursed genomic testing if a formal indication for adjuvant chemotherapy was established by the multidisciplinary team. Patients were excluded from reimbursement meeting the definition of low risk (at least four of the following characteristics: Grade 1, Tumor size ≤ 1 cm, node negative, Ki67 < 15%, ER > 80%) or high risk (at least four of the following characteristics: Grade 3, T3/T4, node positive, Ki67 > 30%, ER < 30%) (Supplementary Table 1). After the approval of genomic testing reimbursement by the Italian National Health System in May 2021, the indication for testing slightly changed to include ER+/HER2- breast cancer cases that are “considered uncertain, when a further assessment of the actual utility of the addition of chemotherapy to adjuvant endocrine therapy is needed.” MGAs available in Italy included Oncotype DX, MammaPrint, EndoPredict, and Prosigna.

### Patients

We conducted a retrospective study including all consecutive patients who were tested with MGAs at six hospitals in Italy between July 2020 and July 2022. In total, 1,133 patients were identified. Given that Oncotype DX was the preferred test in 97% of cases and the sole test used in five of the six centers, we only included Oncotype DX requests in our analysis.

### Procedures

We used anonymized aggregated data obtained from the healthcare services' information systems of the six participating cancer centers. Patients were assigned to low (Recurrence Score, RS, 0–25) or high (RS 26–100) RS category according to the TAILORx and RxPONDER thresholds [20, 21]. The clinicopathologic variables were evaluated by the local pathologists of each center. To evaluate the distribution of RS category according to clinicopathologic features, the following variables were considered: tumor size (T1 vs T2 vs T3), nodal status (N0 vs N1), grade (G1 vs G2 vs G3), Ki67 (< 20% vs 21–30% vs > 30%), and age (≤ 50 years vs > 50 years).

### Study Objectives

We aimed to evaluate the clinicopathologic parameters of the patients who were tested, as well as the distribution of RS category according to these features. In addition, we compared our data with those from the TAILORx and RxPONDER trials [20, 21]. Second, we aimed to examine the trend in test prescription over time, looking at data from four semesters between July 2020 and July 2022. Finally, we aimed to assess whether the clinicopathologic features of tested patients changed over time.

### Statistics

Descriptive statistics were used to summarize patient and tumor characteristics. The association of each clinicopathologic feature and RS category was assessed using Pearsonʼs Chi-Squared Test. All *p*-values were two sided, and statistical significance was set at *p* ≤ 0.05. Since the data were aggregated, only univariate analysis was performed.

### Ethical issues

The study analyzed aggregated data which have been anonymized from previously collected patient information and did not involve any intervention or impact on patient care. The patients had provided informed consent before undergoing the Oncotype Dx test. The study protocol (ONC/OSS-03/2023) was approved by the Ethics Committee of the coordinating institution (IRCCS Humanitas Research Hospital) and followed local regulation and ethical guidelines.

## Results

### Patients

Between July 2020 and July 2022, a total of 1,098 Caucasian patients from six referral centers in Lombardy were tested with Oncotype DX. Among these patients, 642 (58.5%) were older than 50 years old, 655 (59.7%) had tumors ≤ 2 cm, 577 (52.6%) were node negative, 419 (38.2%) had G3 tumors, and 244 (22.2%) had a Ki67 > 30%. Overall, the requests for patients with tumors ≥ 5 cm or with G1 tumors were rare (3.1% and 3%, respectively) (Table 1).

**Table 1:** Distribution of Oncotype DX RS according to clinicopathologic features

Characteristic	All (%)	RS 0–25 (%)	RS 26–100 (%)	*p*-value
*Age*				
≤ 50 y	456 (41.5)	341 (74.8)	115 (25.2)	
> 50 y	642 (58.5)	461 (71.8)	181 (28.2)	0.274
*Tumor size*				
T1	655 (59.7)	488 (74.7)	167 (25.3)	
T2	409 (37.2)	288 (70.4)	121 (29.6)	
T3	34 (3.1)	26 (79.4)	8 (20.6)	0.309
*Nodal status*				
N0	577 (52.6)	377 (65.3)	200 (34.7)	
N1	521 (47.4)	425 (81.6)	96 (18.4)	< 0.001
*Grade*				
G1	33 (3)	31 (93.9)	2 (6.1)	
G2	646 (58.8)	538 (83.3)	108 (16.7)	
G3	419 (38.2)	233 (55.6)	186 (44.4)	< 0.001
*Ki67*				
0–20%	436 (39.7)	377 (86.5)	59 (13.5)	
21–30%	418 (38.1)	311 (74.4)	107 (25.6)	
> 30%	244 (22.2)	114 (46.7)	130 (53.3)	< 0.001
Total	1098 (100)	802 (73)	296 (27)	

The clinicopathologic features of patients who have received a genomic test prescription were significantly different between node-negative and node-positive patients.

**Fig. 1:**
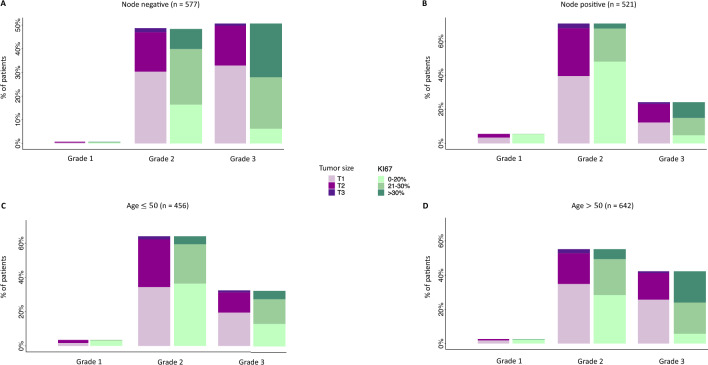
Distribution of Oncotype DX requests according to nodal status and age. Distribution of requests in node-negative (**A**) and node-positive (**B**) patients, and in patients ≤ 50 years (**C**) and > 50 years (**D**)**,** stratified according to tumor size, grade, and Ki67 levels

Genomic test requests for T1, T2, and T3 tumors were 63.6%, 33.8%, and 2.6%, respectively, in the node-negative group, and 55.3%, 41.1%, and 3.6%, respectively, in the node-positive group (*p* = 0.018). Regarding tumor grade, G1, G2, and G3 tumors were 0.7%, 48.5%, and 50.8%, respectively, in the node-negative group, and 5.6%, 70.2%, and 24.2%, respectively, in the node-positive group (*p* < 0.001). Requests for tumors with Ki67 < 20%, Ki67 21–30%, and Ki67 > 30% were 23%, 45.6%, and 31.4%, respectively, in the node-negative group, and 58.2%, 29.8%, and 12%, respectively, in the node-positive group (*p* < 0.001) (Fig. 1A, B).

The distribution of clinicopathologic features of patients who were tested with Oncotype DX was significantly different between age groups, except for tumor size. In patients with 50 years or younger, genomic test requests for T1, T2, and T3 tumors were 55.7%, 41.4%, and 2.9%, respectively, while in patients older than 50 years, they were 62.5%, 34.3%, and 3.2%, respectively (*p* = 0.053). The requests for G1, G2, and G3 tumors were 3.5%, 64%, and 32.5%, respectively, in patients with 50 years or younger, and 2.6%, 55.1%, and 42.2%, respectively, in patients older than 50 years (*p* = 0.004). Tumors with Ki67 < 20%, Ki67 21–30%, and Ki67 > 30% were 44%, 36.4%, and 19.6%, respectively, in patients with 50 years or younger, while in patients older than 50 years, they were 36.6%, 39.3%, and 24.1%, respectively (*p* = 0.032) (Fig. 1C-D).

Interestingly, we found that a significant proportion (42%) of node-positive patients who received a genomic test prescription were 50 years old or younger, which is also similar to the proportion in the node-negative group (41.1%). Overall, patients with node-positive tumors who were 50 years old or younger accounted for approximately 20% of the total population receiving a test prescription.

### Correlation between clinicopathologic features and Recurrence Score

**Fig. 2:**
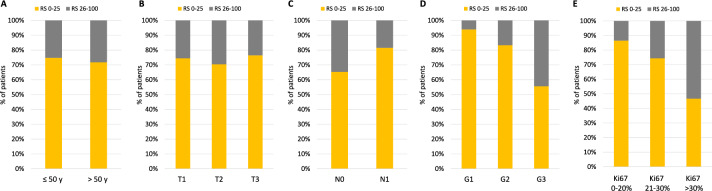
Correlation between Recurrence Score and clinicopathologic features. Recurrence Score distribution in patients stratified according to age (**A**)**,** tumor size (**B**)**,** nodal status (**C**)**,** tumor grade (**D**)**,** and Ki67 levels (**E**)

Overall, the Oncotype DX test identified 803 out of 1,098 patients (73.1%) who had a RS score of 0–25, suggesting potential benefits in terms of chemotherapy sparing and drug costs. The proportion of patients with low RS was similar between age groups (74.8% of patients with 50 years or younger, and 71.8% of patients older than 50 years, respectively, *p* = 0.27), as well as between subgroups stratified by tumor size (74.7%, 70.4%, and 79.4% in patients with T1, T2, and T3, respectively, *p* = 0.22) (Table 1** and **Fig. 2).

There were significant differences in the distribution of RS risk groups when tumors were stratified by grading and Ki67 levels. Specifically, low RS was found in 93.9%, 83.3%, and 55.6% of G1, G2, and G3 tumors, respectively (*p* < 0.001), and in 86.5%, 74.4% and 46.7% of tumors with Ki67 < 20%, Ki67 21–30%, and Ki67 > 30%, respectively (*p* < 0.001) (Table 1 and Fig. 2).

The proportion of patients with low RS in the node-negative group was significantly lower than in the node-positive group (65.7% vs 81.6%, *p* < 0.001) (Table 1 and Fig. 2).

The correlation between clinicopathologic features and RS within node-negative and node-positive patients recapitulated what has been observed in the overall population: in both groups, RS significantly correlated with grade and Ki67 levels but not with age or tumor size (Supplementary Fig. 1).

**Fig. 3:**
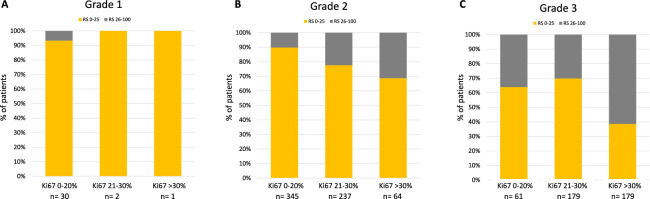
Correlation between Recurrence Score and combined Grade-Ki67. Recurrence Score distribution in Grade 1 (**A**)**,** Grade 2 (**B**)**,** and Grade 3 (**C**) tumors stratified according to Ki67 levels

Of note, despite the significant correlation of RS with grade and Ki67, Oncotype DX identified a significant proportion of patients with clearly poor biological features having a low RS. Among 179 patients with G3 tumors and Ki67 > 30%, 69 (38.6%) had a RS 0–25 (Fig. 3), and this proportion was even higher (50%) within the node-positive patients with these high-risk features (Supplementary Fig. 2). On contrary, among 30 patients with G1 tumors and Ki67 ≤ 20%, 2 (6.7%) had a RS higher than 25 (Fig. 3).

### Temporal trend of test prescription

We observed a progressive increase in genomic testing requests over the two years considered, with the number of requests increasing from 182 between July 2020 and December 2020, to 230 between January 2021 and June 2021, 322 between July 2021 and December 2021, and 364 between January 2022 and June 2022.

The distribution of clinicopathologic characteristics among patients who received a test prescription did not differ significantly between the four semesters, including age, tumor size, nodal status, and Ki67 (Supplementary Table 2). However, there was a trend towards an increase in Oncotype DX requests for node-positive patients after the presentation of RxPONDER trial data. The requests for node-positive patients increased from 41.2% between July 2020 and December 2020 to 49% between January 2021 and July 2022 (*p* = 0.06).

We found a significant variability in the distribution of tumor grade over time. From July 2020 to July 2022, there was a progressive increase in the proportion of patients with G2 tumors who received a test prescription and a corresponding decrease in the proportion of patients with G3 tumors. Specifically, the requests for patients with G2 tumors were 48.9%, 55.2%, 60.2%, and 64.8% in the first to the fourth semester, respectively (*p* = 0.002), while requests for patients with G3 tumors were 47.3%, 41.7%, 36%, and 33.2% in the same period (*p* = 0.007) (Supplementary Table 2).

### Comparison with TAILORx and RxPONDER data

The clinicopathologic characteristics of node-negative patients who underwent genomic testing in our study were significantly different from those of patients included in the TAILORx trial [20].

We tested a higher percentage of patients aged 50 years or younger (41.1% vs 31.4%, *p* < 0.001), a lower percentage of patients with tumors ≤ 2 cm (63.6% vs 74.8%, *p* < 0.001), a higher percentage of patients with G3 tumors (50.6% vs 17.8%, *p* < 0.001), and a lower percentage of patients with G1 tumors (0.7% vs 26.6%, *p* < 0.001) (Table 2). As expected, given the poorer biological characteristics of node-negative patients in our series, the proportion of low RS was also significantly lower than that observed in TAILORx (65.7% vs 85.7%, *p* < 0.001).

**Table 2:** Comparison of clinicopathologic features and RS results of our series with TAILORx and RxPONDER

Characteristic	Lombardy series (N0) (*n* = 577)	TAILORx (*n* = 9719)	*p*-value
*Age*			
≤ 50 y	237 (41.1)	3054 (31.4)	
> 50 y	340 (58.9)	6665 (68.6)	< 0.001
*Tumor size*			
T1	367 (63.6)	7271 (74.8)	
T2	195 (33.8)	2448 (25.2)	
T3	15 (2.6)	0	< 0.001
Grade*			
G1	4 (0.7)	2512 (25.8)	
G2	281 (48.7)	5242 (53.9)	
G3	292 (50.6)	1676 (17.2)	< 0.001
*RS*			
0–25	380 (65.7)	8330 (85.7)	
26–100	197 (34.3)	1389 (14.3)	< 0.001

Characteristic	Lombardy series (N1) (*n* = 521)	RxPONDER (*n* = 5018)	*p*-value
*Age* †			
≤ 50 y	219 (42)	1224 (24.4)	
> 50 y	302 (58)	3794 (75.6)	< 0.001
*Tumor size*			
T1	288 (55.3)	2923 (58.3)	
T2	214 (41.1)	1843 (36.7)	
T3	19 (3.6)	252 (5.0)	0.084
Grade*			
G1	29 (5.6)	1218 (24.3)	
G2	366 (70.2)	3215 (64.3)	
G3	126 (24.2)	507 (10.1)	< 0.001
*RS* ‡			
0–25	425 (81.6)	5083 (83.1)	
26–100	96 (18.4)	1035 (16.9)	0.380

^*^Tumor grade was unknown for some pts in TAILORx and RxPONDER

^†^In RxPONDER, age cut-offs were < 50 and ≥ 50 years old

^‡^In RxPONDER, numbers include all pts screened for the trial (n = 6118)

We also observed significant differences in the distribution of patients age and tumor grade within the node-positive group of patients tested in our series compared to patients enrolled in the RxPONDER trial [21]. We found a higher proportion of node-positive patients who were 50 years of age or younger (42% vs 24.4%, *p* < 0.001), a lower proportion of patients with G1 tumors (5.6% vs 24.7%, *p* < 0.001), and a higher proportion of patients with G3 tumors (24.2% vs 10.3%, *p* < 0.001) (Table 2). However, it should be noted that the distribution of tumor grade in RxPONDER refers to patients enrolled in the trial (therefore, with RS 0–25). Considering the total population screened for RxPONDER, the proportion of node-positive patients with low RS was similar to that observed in our series (83.1% vs 81.6%, *p* = 0.38) (Table 2).

## Discussion

The incorporation of MGAs in clinical practice has profoundly changed the decision-making process for the adjuvant therapy of patients with ER+/HER2- early breast cancer.

Several real-world studies have consistently shown that the use of these signatures can alter treatment recommendation in approximately one-third of patients [9, 11, 17, 22], and there is a large body of evidence supporting the cost-effectiveness of MGAs in various community practice settings [23–25]. As a result, many health systems worldwide have approved the reimbursement of these tests for ER+/HER2- breast cancer patients since 2006, but reimbursement in Italy was not approved until July 2019 in Lombardy [19] and then later countrywide in May 2021.

To the best of our knowledge, this is the first report of the use of MGAs in Italy after reimbursement, and it is the largest real-world study conducted after the availability of data from the large TAILORx, RxPONDER, and MINDACT trials. Our study has uncovered several findings.

First, among the commercially available genomic assays, Oncotype DX resulted by far the preferred test. Prescribing oncologists chose Oncotype DX for 1,098 out of 1,133 patients who received an indication for genomic testing, and Oncotype DX was the only test used in five out of six centers. These findings likely reflect that clinicians perceive the predictive information for chemotherapy benefit provided by Oncotype DX as one of the most relevant aspects driving their choice of assay. They also indicate that prescribing oncologists generally adhere to Guidelines recommendations [26]. Indeed, Oncotype DX is the only test recommended by current Guidelines with a level of evidence and a grade of recommendation of I, A for both node-negative and node-positive postmenopausal patients and for node-negative premenopausal patients [18].

Second, the number of genomic test requests progressively increased throughout the considered time frame. We observed a trend towards an increase in requests for node-positive patients, which accounted for about half of the total requests after RxPONDER results were presented. Moreover, the proportion of requests for patients with G2 tumors progressively increased, while those for patients with G3 tumors progressively decreased. These trends are unlikely to reflect a change of the number of eligible patients or of their baseline characteristics. Rather, they might indicate a learning curve for prescribing oncologists during time. In Italy, the use of Oncotype DX and other MGAs remains quite scattered to date, but these data suggest that Italian physicians are coming to have greater “trust” in genomic test results.

Third, our data confirm that no individual clinicopathologic feature was precisely and robustly predictive of RS category. The significantly lower proportion of patients with a low RS in the node-negative compared to the node-positive group (65.7% vs 81.6%) is not surprising, as node-negative patients in our cohort had poorer biological features (more G3 and more Ki67 > 30%) than node-positive patients. This is most likely due to the indication for reimbursed genomic testing, which was for patients with a formal recommendation for adjuvant chemotherapy. Although RS correlated significantly with grade and Ki67, a substantial proportion of patients with G3 tumors (55.6%) or with Ki67 > 30% (46.7%) had a low RS. Moreover, 39% of patients with both G3 tumors and Ki67 > 30% still had a low RS. According to the International Ki67 in Breast Cancer Working Group recommendations, a Ki67 threshold of 30% or greater could be used to proceed with chemotherapy without the need for more expensive commercial multi-parameter gene-expression assays [27]. However, our findings indicate that a chemotherapy recommendation based solely on high Ki67 values may be at least questionable and suggest that, if available, genomic assays should be employed also in those cases with high Ki67 levels when uncertainty on the chemotherapy benefit still remains. Oppositely, we have found that a low but not negligible proportion (1 out of 15) of patients with biological features indicative of clearly low risk (i.e., G1 and low Ki67) had a high RS and, therefore, may derive a benefit from chemotherapy. Although limited by the small numbers, these data pose the question of how many patients missed the opportunity of the test during the early period (when the indication was for patients with formal recommendation to adjuvant chemotherapy) and corroborate the utility of genomic assays not only for treatment de-escalation but, more in general, for treatment tailoring, especially for those cases that may fall in a “gray zone” for chemotherapy recommendations.

Fourth, we found that a substantial proportion (41.5%) of patients who have received a genomic test prescription were 50 years old or younger. More importantly, almost half of these young patients had node-positive tumors, representing about 20% of the overall population.

These data indicate that, although Guidelines do not recommend the use of Oncotype DX or other genomic assays to tailor adjuvant therapy decisions in node-positive premenopausal patients (or patients ≤ 50 years), the oncologists prescribing Oncotype DX considered the test informative also in this population [26]. The role of age and menopausal status has been quite neglected in the development of Oncotype DX and other MGAs. However, both TAILORx and RxPONDER trials have found significant interaction for outcome between chemotherapy effect and age or menopausal status [21, 28], leading to different interpretations of RS based on these factors.

Some data suggested that among the luminal subtype, tumors arising in young women may be biologically distinct [29, 30]. For example, it was found that tumors of young women showed upregulation of biological processes related to growth factor signaling and downregulation of apoptosis-related genes [29]. More recently, Qing et al. have found that tumors in patients 50 years or younger have lower expression of ER-related genes and higher expression of immune-related genes that may determine higher chemo-sensitivity [30]. Although one might speculate that these biological characteristics could lead to a greater cytotoxic effect of chemotherapy, and some data suggest that ER+/HER2- tumors in younger women may be more responsive to chemotherapy [31], evidence from TAILORx and RxPONDER support the hypothesis that most of the observed chemotherapy benefit in young women with low RS might be due to its endocrine effect of ovarian suppression. In fact, in TAILORx, the chemotherapy benefit observed in women 50 years old or younger was primarily limited to women between 40 and 50 years old and waned in younger and older age groups. Additionally, women between 46 and 50 years old appeared to derive a benefit only before menopause, while postmenopausal women in the same age group did not experience any benefit [28]. In RxPONDER, the only subgroup of premenopausal women who did not seem to benefit from the addition of chemotherapy were those who were 50 years of age or older, namely women who are likely to reach menopause soon, even without chemotherapy [21]. Importantly, the rate of ovarian function suppression for premenopausal women in the endocrine therapy alone arm was limited in both trials, which hinders the full applicability of the results in current clinical practice. This could explain why clinicians in our series used Oncotype DX so often in young women with node-positive tumors. Nonetheless, there is an urgent need for prospective data to fill the knowledge gap in this field.

Strengths of our study include a large sample size and the evaluation of real-world MGAs use in a time frame when results from prospective trials were available. Additionally, the reliability of our histological reports, which were conducted in referral cancer centers by pathologists specialized in breast cancer, adds to the validity of our findings.

However, our study clearly has limitations. For instance, we were unable to capture other clinicopathologic variables such as histological subtype, menopausal status, or progesterone receptor status, and our use of an age cutoff to determine menopausal status may be imprecise. In addition, the reproducibility of certain clinicopathologic features such as Ki67 can be variable across labs, which limits the generalizability of our findings. Furthermore, our use of anonymized aggregated data prevented us from conducting additional analyses, such as the evaluation of age and Ki67 as a continuous variable to correlate their values with continuous RS scores, or the introduction of a pathological stage subtype for T1 tumors (T1a–b vs T1c) to ascertain if any distinctions emerge in the administration of Oncotype based on tumor size. More importantly, we were unable to collect treatment and follow-up information, so we could not estimate the exact amount of chemotherapy sparing in our series.

Nevertheless, several decision-impact studies available in the literature consistently showed significant chemotherapy sparing effect of Oncotype DX [9, 11, 12, 14, 17], and given the initial indication of test prescription in Lombardy, it is likely that also in our series, Oncotype DX use has led to a substantial reduction in chemotherapy use and drug cost, with remarkable benefits for both patients and the healthcare system.

In summary, this study highlights the contemporary use of MGAs in clinical practice and showed that Oncotype DX was by far the preferred test adopted by clinicians. Our findings confirm that individual clinicopathologic features do not robustly predict RS category and suggest that, despite Guidelines recommendation, clinicians perceive Oncotype DX as a potentially useful tool for guiding treatment decisions even in young patients with node-positive disease. Nevertheless, our data cannot support the clinical utility of the test in this context.

### Supplementary Information

Below is the link to the electronic supplementary material.

Supplementary file1 (DOCX 13 kb)

Supplementary file2 (DOCX 19 kb)

Supplementary file3 (PDF 106 kb)

## Data Availability

The data that support the findings of this study are available in form of anonymized aggregated data from the corresponding author upon reasonable request.
