# Supplementary material for: Real-world use of multigene signatures in early breast cancer: differences to clinical trials
Source: Breast Cancer Res Treat. 2024 Jan 24;205(1):39–48. doi: 10.1007/s10549-023-07227-0 (PMC11062950; doi:10.1007/s10549-023-07227-0)
Supplement: Supplementary file 1 — Supplementary file1 (DOCX 13 kb) [file 10549_2023_7227_MOESM1_ESM.docx]

**Supplementary Table 1. Exclusion criteria for reimbursed genomic test.**

| **Low risk (at least four of the following characteristics)** | **High risk (at least four of the following characteristics)** |
| --- | --- |
| G1 | G3 |
| T1 (a-b) | T3-4 |
| Ki 67<15% | Ki 67>30% |
| ER>80% | ER<30% |
| N0 | N + |
