# Supplementary material for: Real-world use of multigene signatures in early breast cancer: differences to clinical trials
Source: Breast Cancer Res Treat. 2024 Jan 24;205(1):39–48. doi: 10.1007/s10549-023-07227-0 (PMC11062950; doi:10.1007/s10549-023-07227-0)
Supplement: Supplementary file 2 — Supplementary file2 (DOCX 19 kb) [file 10549_2023_7227_MOESM2_ESM.docx]

**Supplementary Table 2. Characteristics of the Patients for whom an Oncotype DX was requested throughout the 4 semesters considered**

|  | Jul 2020 –  Dec 2020 | Jan 2021 –  Jun 2021 | Jul 2021 –  Dec 2021 | Jan 2022 –  Jun 2022 |
| --- | --- | --- | --- | --- |
| **Age**  ≤ 50 y  > 50 y | 72 (39.6)  110 (60.4) | 97 (42.2)  133 (57.8) | 137 (42.5)  185 (57.5) | 151 (41.5)  213 (58.5) |
| **Tumor size**  T1  T2  T3 | 103 (56.6)  74 (40.7)  5 (2.7) | 133 (57.8)  88 (38.3)  9 (3.9) | 198 (61.5)  114 (35.4)  10 (3.1) | 220 (60.4)  134 (36.8)  10 (2.7) |
| **Nodal status**  N0  N1 | 107 (58.8)  75 (41.2) | 124 (53.9)  106 (46.1) | 157 (48.8)  165 (51.2) | 189 (51.9)  175 (48.2) |
| **Grade**  G1  G2  G3 | 7 (3.8)  89 (48.9)  86 (47.3) | 7 (3.0)  127 (55.2)  96 (41.7) | 12 (3.7)  194 (60.2)  116 (36.0) | 7 (1.9)  236 (64.8)  121 (33.2) |
| **Ki67**  0-20  21-30  > 30 | 66 (36.3)  73 (40.1)  43 (23.6) | 88 (38.3)  85 (36.9)  57 (24.8) | 128 (39.7)  122 (37.9)  72 (22.4) | 155 (42.6)  137 (37.6)  72 (19.8) |
| **Total** | 182 (100) | 230 (100) | 322 (100) | 364 (100) |
