# Supplementary material for: Real-world use of multigene signatures in early breast cancer: differences to clinical trials
Source: Breast Cancer Res Treat. 2024 Jan 24;205(1):39–48. doi: 10.1007/s10549-023-07227-0 (PMC11062950; doi:10.1007/s10549-023-07227-0)

Supplementary Figure 1. Correlation between clinicopathologic features and Recurrence Score in node-negative and node-positive patients. Recurrence Score distribution in node-negative **(A-D)** and node-positive **(E-H)** patients stratified according to age **(A, E)**, tumor size **(B, F)**, tumor grade **(C, G)** and Ki67 levels **(D, H)**

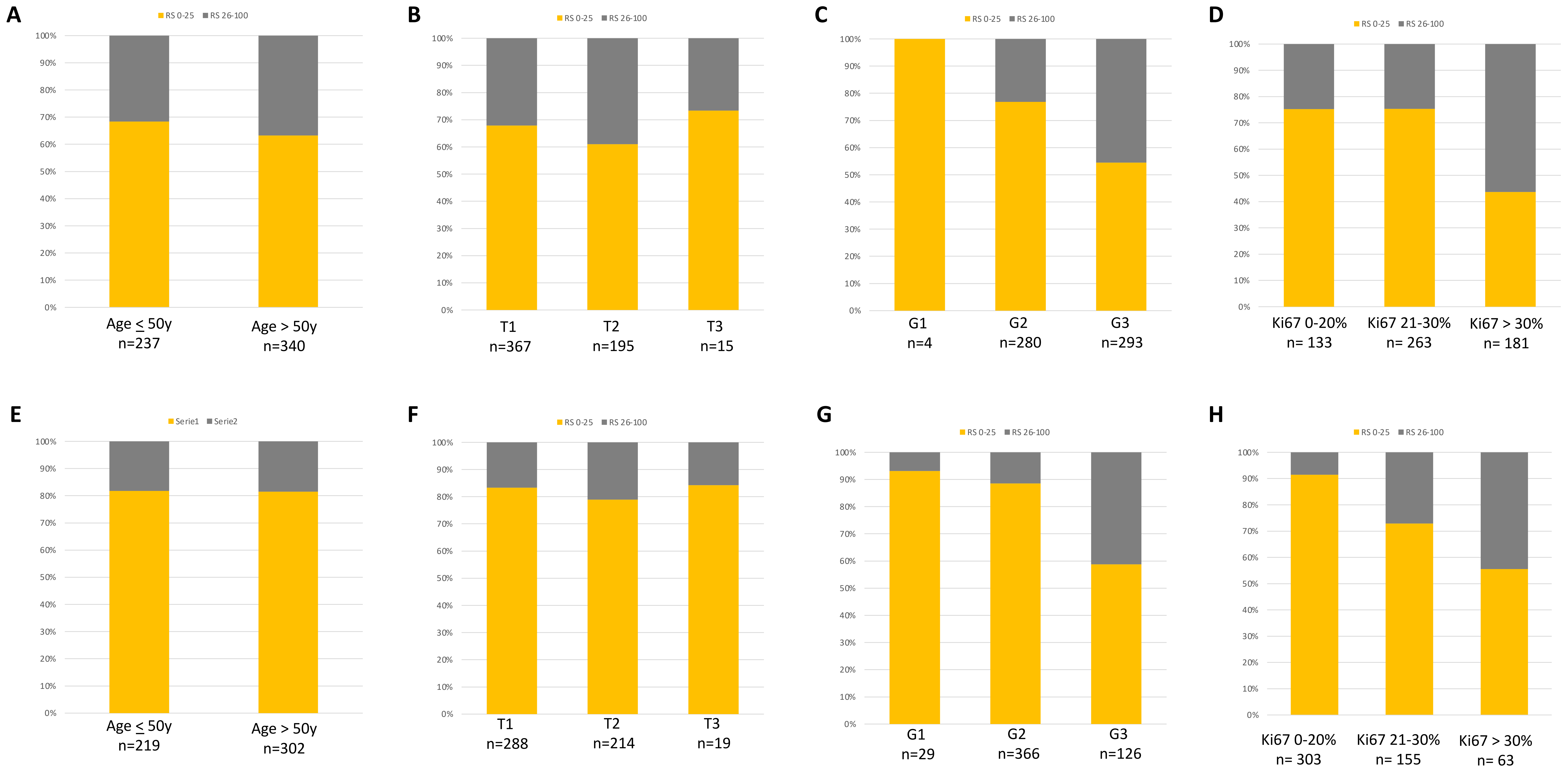

Supplementary Figure 2. Correlation between Recurrence Score and combined Grade-Ki67 in node-negative and node-positive patients. Recurrence Score distribution in node-negative **(A-C)** and node-positive **(D-F)** patients stratified according tumor grade and Ki67 levels

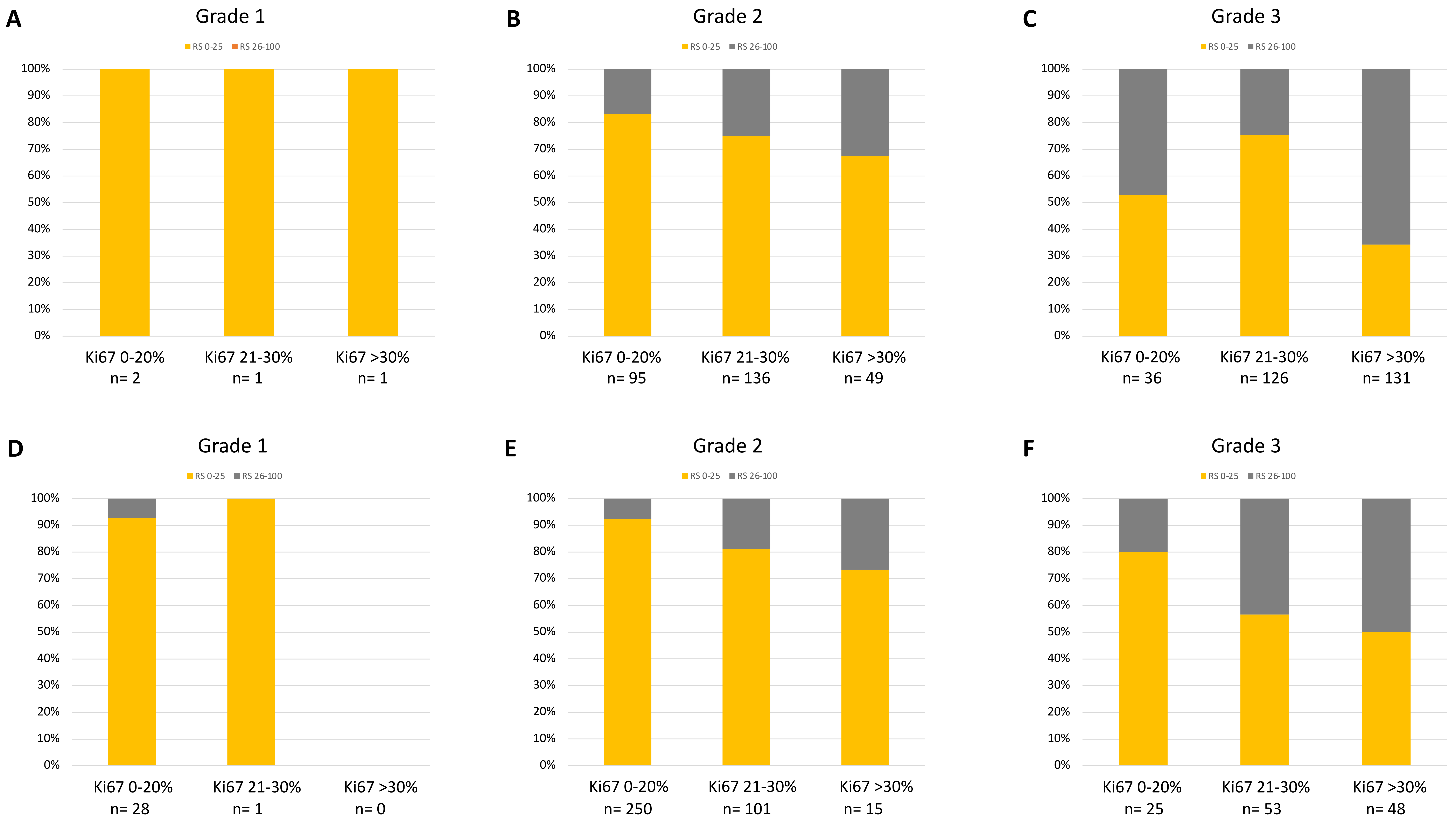

Supplement: Supplementary file 3 — Supplementary file3 (PDF 106 kb) [file 10549_2023_7227_MOESM3_ESM.pdf]
